# Harmonized geospatial data to evaluate the Electric Distribution Networks in the US Northeast

**DOI:** 10.1038/s41597-025-06465-9

**Published:** 2025-12-19

**Authors:** Bernat Salbanya, Jordi Nin, Ramon Gras Alomà

**Affiliations:** 1 TBS Business School, Toulouse, Occitanie France; 2 Universitat Ramon Llull, ESADE, Barcelona, Catalonia Spain; 3 Harvard University, SEAS, Cambridge, Massachusetts USA

**Keywords:** Energy management, Energy justice

## Abstract

Reliable, open-access data on electric distribution networks is crucial for advancing energy equity, enhancing infrastructure resilience, and informing policy evaluation. In this work, we present a harmonized geospatial dataset for the electric distribution networks in the US Northeast, covering Connecticut, Maine, Massachusetts, New Hampshire, New York, Rhode Island, and Vermont. The dataset integrates technical, spatial, and topological data extracted from utility hosting capacity maps (public GIS layers reporting feeder-level estimates of distributed energy resources) and processed using a reproducible pipeline. Our network comprises 3,884,698 line segments, achieving a population coverage of 72.46% and geographic coverage of 84.96%. By bridging complex network theory with spatial infrastructure mapping, this dataset enables a multidimensional assessment of electric grid performance, sustainability, and equity. It allows researchers and policymakers to explore the links between urban and economic development patterns, network morphology, and energy outcomes.

## Background & Summary

Electric distribution networks are critical to delivering power from substations to end users, supporting everyday electricity consumption, and enabling broader energy transitions. Although less visible than transmission infrastructure, distribution systems represent the bulk of grid investments and serve as the primary interface between utilities and consumers^1^. As policy goals increasingly prioritize decarbonization, electrification, and integration of distributed energy resources (DER), the reliability, efficiency, and spatial equity of distribution networks have been subjected to increased scrutiny^2,3^.

In the US Northeast (Connecticut, Maine, Massachusetts, New Hampshire, New York, Rhode Island, and Vermont), a region marked by aging infrastructure, diverse urban morphologies, and high load density, distribution networks face operational constraints intensified by climatic variability, urban-rural disparities, and growing electrification demand^4^. This geography includes major urban centers and rural areas, accounts for roughly one-tenth of the US population, and has ambitious state decarbonization policies, making it a strategic testbed for distribution planning and equity analysis. Despite its importance, open and harmonized medium-voltage distribution datasets that span multiple utilities in this region have been limited; the present release addresses this gap by providing a cleaned, standardized and tract-linked layer suitable for comparative and equity-focused studies. Integrating rooftop solar, electric vehicles, and building electrification into this legacy infrastructure requires better visibility into network capacity, topology, and community-level vulnerability^5,6^. As these challenges grow, planning tools must evolve from traditional static methods to data-rich, spatially explicit, and interdisciplinary frameworks. While such integrated, spatially grounded approaches are well-established in fields like urban planning, economics, innovation, and mobility^7^, they remain underdeveloped in the context of electric distribution systems, highlighting a critical research gap this work begins to address.

This work presents a new harmonized dataset and analytical framework that integrates complex network theory and geospatial topology to evaluate the spatial structure of electric distribution networks in the US Northeast. By modeling the grid as a graph with nodes (e.g. substations) and edges (e.g. feeders), we extract topological features, such as centrality, density, and fractality, that shape system performance and vulnerability^8,9^. In practice, high edge betweenness centrality flags feeder segments whose outage would sever many shortest paths (bottlenecks), whereas higher intersection density and average degree indicate greater redundancy and shorter detours during contingencies, supporting continuity of service. Likewise, low orientation entropy and low fractality tend to reflect radial layouts that concentrate flows and are more sensitive to single contingencies, while higher entropy and fractality indicate more meshed structures that distribute load and offer alternative routing, informing resilience upgrades and restoration planning. Although the sociodemographic data are not included in this dataset, the use of the US Census geographic identifiers (GEOIDs) (a unique 12-digit codes assigned to each census tract) enables seamless merging with external sources, supporting tract-level analysis of income, housing, and energy burden to identify structural inefficiencies and inequities in service delivery^10^.

This resource enables new analyses that were previously difficult due to the lack of harmonized, open medium-voltage data: (i) tract-level studies of network morphology, accessibility, and infrastructure equity by linking topology to Census GEOIDs; (ii) comparative benchmarking across utilities and states using standardized fields (hosting capacity, operating voltage, circuit rating); (iii) resilience and criticality assessments with graph measures such as betweenness, closeness, straightness, entropy, and fractality; and (iv) siting and screening of distributed energy resources using a consistent, multi-state spatial layer. Our contribution integrates and cleans hosting capacity layers across seven states, standardizes schemas, assigns Census tract identifiers, and publishes both edge-level geometries and tract-level metrics with a reproducible Python pipeline and persistent DOIs.

The dataset merges utility-hosted hosting capacity maps and spatial infrastructure layers, and aligns them with census tract geometries via spatial join, offering a reproducible foundation for analyzing electricity distribution as a socio-technical system. It builds on recent work that emphasizes open and harmonized geospatial data for energy system planning and infrastructure layout^11,12^. By advancing a scalable methodology to analyze distribution networks, this work supports the design of equitable, data-informed strategies for a just and resilient energy transition^13,14^.

## Methods

This section details the complete computational workflow used to clean, standardize, and analyze electric distribution circuit data across multiple US states in the Northeast region. The process integrates raw geospatial data from utility-hosted Hosting Capacity Maps, performs technical and spatial transformations, and produces harmonized data sets suitable for network science and equity analysis. All data sources, preprocessing steps, and computations are described below to enable full reproducibility.

### Step 1 - Data Sources

Circuit-level data were sourced from public utility hosting capacity maps for Connecticut, Massachusetts, Maine, New Hampshire, New York, Rhode Island, and Vermont. These states present a mix of urban, suburban, and rural settings, offering a diverse testbed to evaluate the relationship between the grid structure and socioeconomic conditions. We used publicly available geospatial datasets from utility-hosted Hosting Capacity Maps to derive the physical structure of the electric distribution grid. Hosting Capacity Maps refer to public, utility-maintained GIS layers that summarize circuit attributes and report an estimate of how much additional distributed energy resource (DER) capacity a feeder segment can accommodate under normal operating conditions without requiring distribution upgrades. These maps provide polyline geometries for electric feeders and circuits, along with tabular attributes such as substation name, hosting capacity (MW), operating voltage (kV), and circuit rating (A). Datasets were downloaded as a FeatureService (ArcGIS REST endpoint) and parsed using the requests and geopandas Python libraries. Datasets were provided in a different geospatial format (ESRI Shapefile). Polylines were cleaned to remove duplicates and invalid geometries. Only segments within the bounding geometry of the US Northeast were retained. All geometries were reprojected to WGS84 / World Geodetic System (EPSG:4326) for area-preserving spatial analysis. The spatial resolution of our study is at the census tract level, providing fine-grained demographic and geographic detail. Census tract geometries were obtained from the US Census Bureau’s TIGER/Line shapefiles. Table 7 lists the utilities, states, and the exact ArcGIS REST endpoints used to retrieve hosting capacity data for this study. Each URL was accessed in June 2025^15–29^.

Due to the large size of the raw hosting capacity datasets, we do not host the raw data directly in the GitHub repository. Instead, the main script downloads the harmonized datasets from Harvard Dataverse (DOIs listed in the Data Records section) and processes them for analysis.

### Step 2 - Data Cleaning and Standardization

A set of state-specific cleaning functions was developed to process each dataset according to its structure. These functions applied a common logic: renaming columns for consistency, dropping redundant fields, handling missing values, and tagging data with utility names and metadata. The data cleaning and standardization process was critical to harmonizing the circuit datasets obtained from multiple service providers. Each provider reported electric line data in a different schema, with different naming conventions, units, data quality, and missing fields. We implemented utility-specific cleaning functions held in process_data.py that performed the following steps:

#### Column Renaming and Dropping

For each utility, a custom rename_map was applied to unify field names such as hosting capacity (HostCap_MW), voltage (Voltage_kV), phase configuration (Phase), and circuit rating (CircRat_A). Irrelevant or redundant fields (e.g. metadata, aliases, unused capacity fields) were removed.

#### Geometry and CRS Harmonization

All datasets were re-projected to EPSG:4326 for consistency. Invalid or non-LineString geometries were dropped. When necessary (e.g., Vermont grid), a geometry adjustment function (adjust_geometry) ensured visual consistency and spatial overlay compatibility. This function scaled and translated circuit geometries to fit within specified bounding boxes, such as Vermont’s spatial extent. This function used affine transformations (shapely.affinity) and ensured that the geometries were projected to EPSG:4326 before the transformation.

#### Phase and Voltage Normalization

Phase values were highly inconsistent (e.g., *AB*, *ABCN*, *1*, *3*). We applied a comprehensive phase_mapping dictionary to standardize these to numerical values (*1*, *2* or *3*). Voltage fields were often text-formatted or included erroneous null indicators (e.g., *0*, *240/120 Volts*), which we recoded using a voltage cleaning map and converted to numeric kilovolts (kV).

#### Engineering Calculations

When voltage or current was missing, it was estimated using electrical engineering formulas, assuming a default power factor of 0.95 and a known phase configuration. Using amps_to_voltage(), voltage was estimated from hosting capacity in MW, and circuit rating in A. We assumed the power factor to be 0.95 and the phase configuration (1- or 3-phase systems). The calculate_voltage() function was applied row-wise and rounded the results to a set of known industry standard voltage levels. For records missing current data, the calculate_circuit_rating() function used the reverse formula via mw_to_amps() to calculate circuit rating (A) from hosting capacity and voltage. Apparent power values in kVA were converted to active power in MW using kva_to_mw(), and to current in amps using kva_to_amps() with appropriate phase adjustments. A complete summary of the equations used for these estimations is provided in Table 4.

#### Outlier Handling and Imputation

Extreme values for CircRat_A were capped at the 95th percentile within each dataset to prevent statistical metrics skewness. The missing or zero values for voltage and current were imputed using the mode or filled by group-wise forward and backward imputation.

### Step 3 - Building the US Northeast Electric Network Dataset

#### Merging and Spatial Join

The clean utility datasets were merged into a single GeoDataFrame. Multiple datasets per state or utility were independently cleaned and then merged using merge_and_save(). In addition, spatial_join_with_cen sus() joined the circuit dataset with US Census Tract geometries using spatial predicates (intersects) via geopandas. sjoin(). This enables geographical identification to be assigned to each segment of the electric line, facilitating linkage to external sociodemographic datasets. All columns were cut to 10 characters to ensure compatibility with the ESRI Shapefile format during export. The types were explicitly cast (float64 for MW, A, kV; int for phase). The final data sets were saved in .shp format. All these functions are executed by process_data.py.

#### Network Modeling for Electric Distribution Systems

We modeled the electric grid as a spatial graph *G* = (*V*, *E*), where *V* are nodes, namely intersection points, substations and endpoints; *E* are edges representing the distribution lines between nodes. To convert the polyline dataset into a topological network, we used momepy.gdf_to_nx() with a primal graph approach. Line endpoints and intersections were extracted using Shapely geometry tools and stored as nodes. Each edge was enriched with attributes, including physical length, voltage level, and capacity. We computed comprehensive network morphology metrics for each census tract by spatially overlaying tract geometries onto the graph and aggregating local statistics. This dataset includes a set of morphological and topological metrics designed to capture the structural complexity, connectivity, and operational efficiency of electric distribution networks. The following metrics were computed using the networkx, momepy, igraph, and custom functions implemented in network_metrics.py.

- **Number of Nodes and Edges:** The number of nodes (*N*) and edges (*E*) are direct counts of graph elements: $N=| {\mathcal{V}}| \,;\,E=| {\mathcal{E}}|$. These values serve as scale indicators and are used to compute other normalized metrics.
- **Node Degree**(*k*): The number of connections (edges) associated with a node. The average node degree is: $\overline{k}=\frac{1}{N}{\sum }_{i=1}^{N}\,{k}_{i}.$ A proxy for node centrality and connectivity.
- **Node Strength**(*s*): Sum of edge weights (here, hosting capacity in MW) connected to a node: ${s}_{i}={\sum }_{j\in {\mathcal{N}}(i)}{w}_{ij}$ Reflects the load capacity handled by each node.
- **Total Node Degree and Strength:** The total degree and strength are the sums of node degrees and hosting capacity weights, respectively, across the entire subgraph: $Total\,Degree={\sum }_{i=1}^{N}{k}_{i}\,;\,Total\,Strength={\sum }_{i=1}^{N}{s}_{i}$. These provide aggregate measures of connectivity and load distribution within each tract.
- **Graph Density**(*ρ*): Defined as the ratio of actual edges *E* to the maximum number of possible edges in an undirected graph with *N* nodes: $\rho =\frac{2E}{N(N-1)}.$ Higher density indicates a more interconnected network.
- **Assortativity Coefficient**(*r*): Measures the correlation between degrees of connected nodes: $r=\frac{{\sum }_{i}{j}_{i}{k}_{i}-{[{\sum }_{i}\frac{1}{2}({j}_{i}+{k}_{i})]}^{2}}{{\sum }_{i}\frac{1}{2}({j}_{i}^{2}+{k}_{i}^{2})-{[{\sum }_{i}\frac{1}{2}({j}_{i}+{k}_{i})]}^{2}}$. Indicates whether nodes tend to connect to similar or dissimilar nodes in terms of degree.
- **Average Shortest Path Length** (*L*): The average number of steps along the shortest paths for all possible pairs of nodes: $L=\frac{1}{N(N-1)}{\sum }_{i\ne j}d(i,j).$ Lower *L* suggests higher network efficiency and accessibility.
- **Average Segment Length** ($\overline{l}$): Mean length of circuit line segments: $\overline{l}=\frac{1}{M}{\sum }_{i=1}^{M}\,length({e}_{i}).$ Provides a spatial measure of network granularity.
- **Connectivity:** Defined as the number of connected components in the graph. A fully connected network has connectivity equal to 1.
- **Average Intersection Connectivity:** The ratio of nodes to edges, used as a normalized proxy for connectivity: $Avg.\,Intersection\,Connectivity=\frac{N}{E}$. Lower values may indicate longer feeder lines with fewer branching points, while higher values suggest more distributed infrastructure.
- **Density of Intersections:** Defines how many network nodes exist per unit of land area (km^2^) within a census tract: $Intersection\,Density=\frac{N}{A}$, where *A* is the land area of the tract (from census geometry). Higher density may suggest greater infrastructure access or redundancy.
- **Density of Segments:** Defines the number of edges (line segments) per unit area: $Segment\,Density=\frac{E}{A}$. Higher values may reflect more granular or fine-grained distribution networks.
- **Betweenness Centrality** (*BC*): For a node *v*, betweenness centrality is defined as: $BC(v)={\sum }_{s\ne v\ne t}\frac{{\sigma }_{st}(v)}{{\sigma }_{st}}$, where *σ*_*st*_ is the total number of shortest paths from *s* to *t*, and *σ*_*st*_(*v*) is the number of those paths passing through *v*.
- **Closeness Centrality:** Measures how close a node is to all other nodes in the network: $C(i)=\frac{1}{{\sum }_{j}d(i,j)}$. Two variants were computed: global closeness and local closeness within a 50-meter radius.
- **Straightness Centrality:** Quantifies the directness of paths from node *i* to all other nodes: $S(i)=\frac{1}{N-1}{\sum }_{j\ne i}\frac{{d}_{E}(i,j)}{{d}_{G}(i,j)}$, where *d*_*E*_ is Euclidean distance and *d*_*G*_ is graph distance.
- **Entropy of Edge Orientations** (*H*): Measures the level of directional disorder in the layout of the network. $H=1-{(\frac{{H}_{0}-\log (2)}{\log (n)-\log (2)})}^{2},\,where\,{H}_{0}=-{\sum }_{i=1}^{n}\,{p}_{i}\log ({p}_{i})$, *p*_*i*_ is the proportion of edges in each orientation bin. High entropy suggests more irregular, disorganized grid layouts.
- **Fractal Dimension** (*D*_*f*_): Estimates the self-similarity and hierarchical complexity of the grid using the box-counting method. ${D}_{f}=-\frac{d\,\log \,N(\epsilon )}{d\,\log \,\epsilon }$, where *N*(*ϵ*) is the number of occupied boxes of size *ϵ*. Higher *D*_*f*_ implies more intricate and self-similar patterns.

## Data Records

The dataset is available at US Electric Distribution Networks Dataverse^30–33^. All data used in this study are publicly archived in the Dataverse under four dedicated repositories:

- **US Northeast Electric Circuits**^31^: contains the raw circuit-level data as downloaded from the utility hosting capacity maps.
- **US Northeast Cleaned Data**^30^: contains the cleaned, harmonized, and merged dataset of the US Northeast electric distribution network, including the integrated shapefile US_Northeast_Electric_Network.shp.
- **US Northeast Census Tracts**^33^: contains the US Census tract geometries used as the unit of analysis for network metrics.
- **US Northeast Results**^32^: contains the network metrics aggregated by census tract (GEOID) produced from our analyses.

**Fig. 1:**
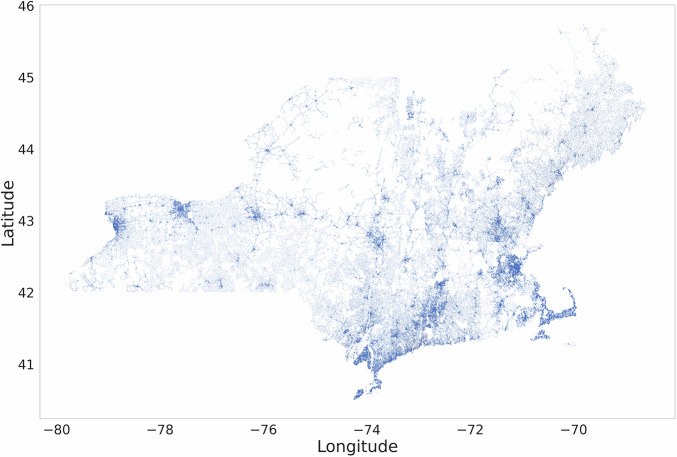
US Northeast Electric Distribution Network. Polylines depict cleaned edges (feeder segments derived from utility hosting capacity layers after harmonization) and nodes (intersections, substations, endpoints).

This repository structure ensures that users can clearly distinguish between raw, cleaned, spatial reference, and analysis-result datasets, thereby improving reproducibility and transparency. The results repository comprises three components: the **network edge geometries,** **node-level metrics**, and **census-tract-level global indicators**. All files are in open formats (.shp for spatial data and .csv for tabular data) and stored in a public repository with persistent DOI. The compiled representation of US Northeast Electric Distribution Network is shown in Fig. 1.

### Electric Circuit Segments (US_Northeast_Electric_Network.shp)

This shapefile stored in the US Northeast Cleaned Data repository^30^, contains line geometries representing medium-voltage electric distribution segments in the US Northeast. It contains 3,884,698 line records, covering 72.46% of the regional population and 84.96% land area. Each record corresponds to a circuit segment described with the key attributes presented in Table 1. These segments represent parts of the electric distribution network and are recorded as spatial lines (geometry). The HostCap_MW field, or hosting capacity, refers to the maximum amount of distributed energy that the circuit segment can accommodate without triggering system upgrades, measured in megawatts. The Voltage_kV indicates the nominal operating voltage of the primary distribution feeder (medium-voltage). Low-voltage secondary networks and transmission-level voltages are not included in this dataset. CircRat_A refers to the circuit’s current-carrying capacity, measured in amperes (A), reflecting how much electric current can flow safely through the segment. The Phase attribute denotes the number of alternating current (AC) phases, typically 1, 2, or 3, which defines the configuration of energy flow. Additional contextual information includes the utility provider responsible for the segment (Company), the US state in which the segment is located (State), and any associated electrical substation (Substation). Finally, each segment is linked to a specific census tract through a unique identifier (GEOID), allowing for integration with demographic or economic data. Together, these fields provide a comprehensive description of the technical and spatial characteristics of the electric distribution network.

**Table 1:** Attributes collected in the US Northeast Electric Network dataset, their data type, brief descriptions, and units.

Field	Type	Description
geometry	LineString	Geometric representation of the circuit segment
HostCap_MW	float64	Hosting capacity of the segment in megawatts (MW)
Voltage_kV	float64	Operating voltage in kilovolts (kV)
CircRat_A	float64	Circuit rating in amperes (A)
Phase	int	Number of phases (1, 2, or 3)
Company	string	Utility company operating the segment
State	string	US state of the segment
Substation	string	Associated substation (if available)
GEOID	string	Census tract identifier assigned via spatial join

### Node-Level Topological Indicators ({GEOID}_node_metrics.csv)

Each GEOID_node_metrics.csv file in the US Northeast Results repository^32^, contains the node-level topological indicators that quantify the structural role and connectivity of each network node within a census tract, including degree, centrality, and clustering measures. Table 2 lists the computed node-level attributes for each node of the electric distribution network graph. These metrics describe the topological role of each node, such as its number of connections (degree), centrality within the network, and proximity to other nodes, providing insights into the structural importance and accessibility of different network junctions, substations, or transformers. Each variable is expressed numerically and contributes to understanding the network’s efficiency, robustness, and flow dynamics at the local scale.

**Table 2:** Attributes collected in each GEOID nodes dataset, their data type, and brief descriptions.

Field	Type	Description
GEOID	string	Census tract identifier
nodeID	string	Unique node identifier
degree	float64	Number of connected edges
nodes_betweenness	float64	Betweenness centrality
clustering_coefficient	float64	Local clustering coefficient
closeness400	float64	Local closeness centrality (radius = 50)
closeness_global	float64	Global closeness centrality
straightness_centrality	float64	Straightness centrality

### Census Tract-Level Metrics ({GEOID}_global_metrics.csv)

These files contain aggregated metrics that describe the structural complexity, connectivity, and spatial organization of the electric distribution network within each census tract, enabling comparative analysis in geographical contexts. Table 3 presents the set of aggregated network metrics computed for each US Census Tract (identified by GEOID) in the dataset. These global indicators summarize the structural, topological, and spatial properties of the electric distribution network within each tract. The variables include basic scale measures (e.g. number of nodes and edges), average and total connectivity, load statistics, and advanced descriptors such as assortativity, betweenness, closeness, straightness, entropy, and fractal dimension. Together, these metrics provide a comprehensive characterization of the morphology, efficiency and robustness of the local grid, enabling spatial comparisons and integration with demographic or energy burden data. These records, stored in the US Northeast Results repository^32^, are designed to support comparative analysis with external socioeconomic indicators, although such data are not included in this dataset.

**Table 3:** Attributes collected in each GEOID global metrics dataset, their data type, brief descriptions, and units.

Field	Type	Description
GEOID	string	Census Tract identifier
number_edges	int	Total number of edges in the tract
number_nodes	int	Total number of nodes in the tract
total_node_degree	float64	Sum number of edges per node
total_node_strength	float64	Sum weighted node strength (by hosting capacity)
avg_node_degree	float64	Average number of edges per node
avg_node_strength	float64	Average weighted node strength (by hosting capacity)
density	float64	Graph density
density_of_intersections	float64	Number of nodes per unit area
density_of_segments	float64	Number of edges per unit area
assortativity	float64	Average nodes degree assortativity
avg_shortest_path	float64	Average shortest path length
avg_segment_length	float64	Average segment length in meters
connectivity	int	Number of connected components
avg_intersection_connectivity	float64	Average intersection connectivity
avg_nodes_betweenness_centrality	float64	Average nodes betweenness centrality
avg_edges_betweenness_centrality	float64	Average edge betweenness centrality
avg_global_closeness	float64	Average global closeness centrality
avg_local_closeness	float64	Average local closeness
avg_straightness_centrality	float64	Average straightness of node paths
entropy	float64	Orientation entropy of edge directions
fractality	float64	Estimated fractal dimension of topology

### Suitability for Power Systems Analyses

The dataset presented here provides a high-resolution geospatial representation of electric distribution circuits in the US Northeast, including line geometries, hosting capacity, operating voltage levels, and circuit ratings on the census tract scale. These attributes make the data set particularly suitable for analyses of network morphology, spatial accessibility, infrastructure equity, and resilience. However, to perform full power flow simulations, dynamic stability studies, or protection coordination analyses, additional data would be required. Such data include time-resolved load and generation profiles, transformer impedances, protective device settings, voltage regulation equipment details, and switching states. When combined with these operational and engineering parameters, the present data set could serve as a foundational spatial layer for advanced power system modeling and planning.

## Technical Validation

To ensure the accuracy, completeness, and representativeness of the dataset, we implemented a multi-tier validation process involving statistical tests, spatial coverage checks, and cross-comparison with external data sources.

### Internal Consistency and Network Connectivity

We validated topological correctness by verifying that all edges form valid LineStrings and that all graphs are weakly connected within each census tract. To ensure that the modeled grid reflected the actual physical connectivity of the electric distribution system, we used the momepy.gdf_to_nx() function to convert the cleaned line geometries into a topological graph. This function detected line intersections and created nodes at shared endpoints within a defined snapping tolerance, ensuring accurate connectivity between adjacent segments. Network connectivity and average path length were calculated for each subgraph to confirm the existence of plausible network structures. Cases where disconnected components were identified (e.g. due to data gaps) were flagged in metadata and excluded from global metric aggregation. The excluded data represented 2.3% of the original LineStrings.

### Spatial Coverage Verification

We performed a spatial overlay of the cleaned network against US Census TIGER/Line shapefiles to determine coverage per tract. The dataset covers approximately 72.46% of the regional population and 84.96% land area in the study region, with some rural tracts having incomplete utility coverage due to data availability. These statistics were computed using population- and area-weighted aggregation across all states.

**Table 4:** Engineering equations for estimating electric circuit variables from available attributes.

Estimated Variable	Equation	Description
Voltage (V); three-phase	$V=\frac{P\cdot 1{0}^{6}}{\sqrt{3}\cdot I\cdot PF}$	Estimate line-to-line voltage from hosting capacity (MW) and current (A).
Voltage (V); single-phase	$V=\frac{P\cdot 1{0}^{6}}{I\cdot PF}$	Single-phase version of voltage estimation.
Current (A); three-phase	$I=\frac{P\cdot 1{0}^{6}}{\sqrt{3}\cdot V\cdot PF}$	Estimate current from hosting capacity (MW) and voltage (V).
Current (A); single-phase	$I=\frac{P\cdot 1{0}^{6}}{V\cdot PF}$	Single-phase version of current estimation.
Power (MW); three-phase	$P=\frac{\sqrt{3}\cdot V\cdot I\cdot PF}{1{0}^{6}}$	Compute real power (MW) from voltage and current.
Power (MW); single-phase	$P=\frac{V\cdot I\cdot PF}{1{0}^{6}}$	Single-phase version of real power computation.
Apparent Power (kVA)	$S=\frac{P}{PF}\cdot 1{0}^{3}$	Convert real power (MW) to apparent power (kVA).
Current from kVA; three-phase	$I=\frac{S\cdot 1{0}^{3}}{\sqrt{3}\cdot V}$	Estimate current from apparent power (kVA) and voltage (V).
Current from kVA; single-phase	$I=\frac{S\cdot 1{0}^{3}}{V}$	Single-phase version of current-from-kVA computation.

Assumes a power factor (PF) of 0.95 unless otherwise noted.

**Fig. 2:**
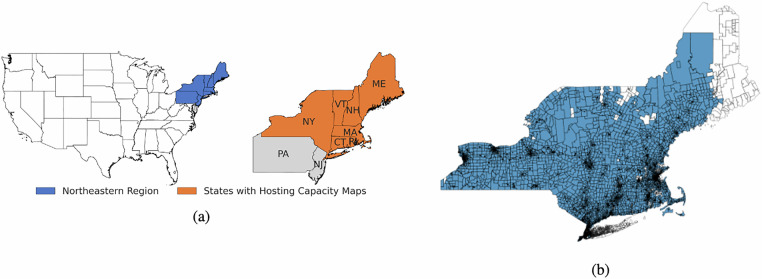
Study area and census-tract coverage. (**a**) Continental United States with the US Northeast study region highlighted. (**b**) Spatial coverage of the dataset at the census-tract level: tracts with network data after cleaning and merging are shaded in blue; tracts without network data are shown in white.

To assess spatial completeness, we calculated the percentage of population and land area covered by the dataset at the state and utility level. Coverage varies substantially across regions: urban and suburban areas generally exhibit near-complete data availability, while rural tracts show more frequent gaps. These gaps are primarily due to certain local utilities that do not publish Hosting Capacity Maps, or do so only in non-standardized formats incompatible with our pipeline. Table 5 summarizes spatial coverage by utility, and Fig. 2 illustrates the completeness of the tract level in the Northeast area of the United States.

**Table 5:** Electric Distribution Data Coverage by State and Utility.

State	Utilities	Population Coverage	Land Area Coverage
Connecticut	Avangrid, Con Edison, Eversource, National Grid, United Illuminating (Avangrid)	97.7%	99.1%
Maine	Avangrid (CMP), Eversource	82.21%	57.82%
Massachusetts	Avangrid, Eversource, Green Mountain Power, Liberty Utilities, National Grid, Unitil	91.6%	94.54%
New Hampshire	Avangrid (CMP), Eversource, Green Mountain Power, Liberty Utilities, National Grid, Unitil	99.45%	96.07%
New York	Avangrid, Central Hudson, Con Edison, Eversource, Green Mountain Power, National Grid, Orange And Rockland	58.37%	97.16%
Rhode Island	Eversource, National Grid	99.91%	99.16%
Vermont	Avangrid, Eversource, Green Mountain Power, Liberty Utilities	82.1%	87.02%

### Company-by-Company Validation

To ensure reproducibility and geographic generalizability, all analyzes were repeated for each individual state. This included recomputation of PCA components, re-aggregation of network metrics, and revalidation of correlations. The fit quality was evaluated using adjusted R^2^ and Shapiro-Wilk tests on the residual distributions. Log transformations were applied on skewed variables to meet the assumptions of the linear model.

### Visual and Statistical Review

Histograms, boxplots, and correlation matrices were generated for all key attributes (e.g. hosting capacity, circuit rating, entropy) to inspect distributional properties and potential biases. The results were consistent with expected values in urban vs. rural tracts. Furthermore, entropy and fractality scores showed spatial clustering patterns aligned with known grid morphology, further supporting the validity of the dataset structure.

### Hosting Capacity Distributions

**Fig. 3:**
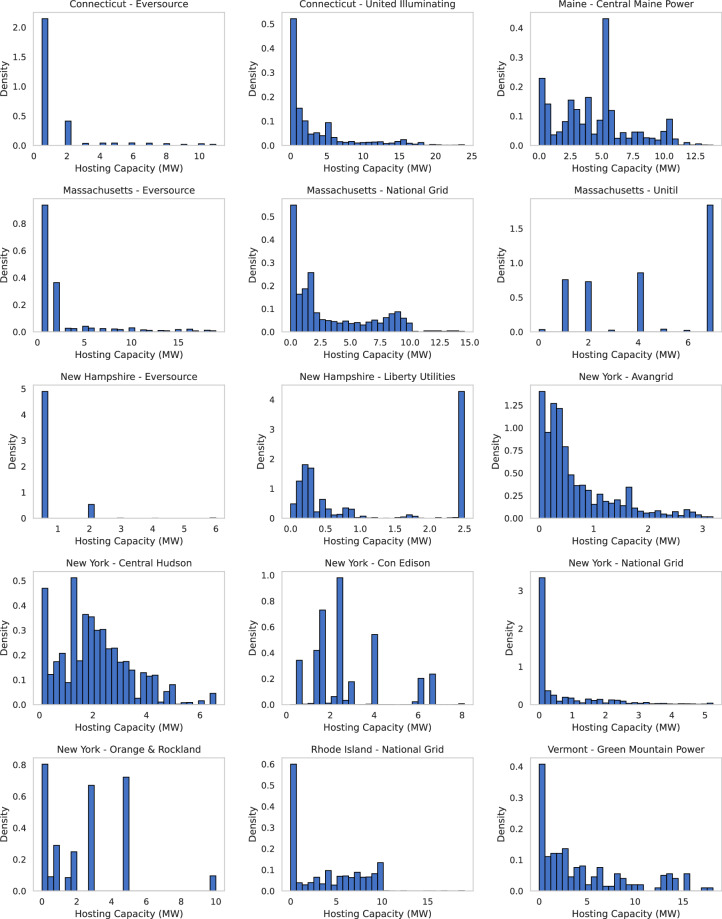
Hosting Capacity (MW) histograms by utility operating company.

Figure 3 presents histograms of the hosting capacity values (in MW) between circuit segments for each utility in the dataset. A clear heterogeneity is observed between utilities: urban and suburban utilities such as ConEdison and Eversource exhibit broader, right-skewed distributions, with many segments supporting higher capacities. In contrast, rural-focused utilities like Green Mountain Power or Liberty Utilities display narrower, more concentrated distributions at lower capacity values. These patterns reflect the structural and load diversity of the electric infrastructure in the service territories and can inform the equitable integration planning of the DER.

### Operating Voltage Distributions

**Fig. 4:**
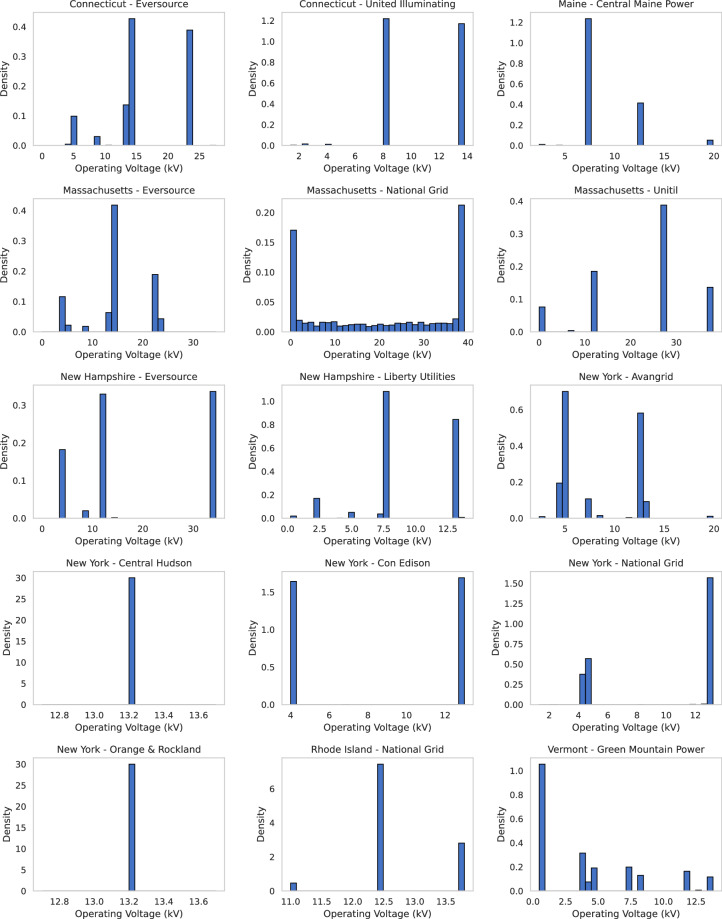
Operating Voltage (kV) histograms by utility operating company.

Figure 4 shows the distribution of operating voltages (in kV) across circuit segments by utility. Most utilities display discrete modal peaks at standard engineering values, such as 4.16 kV, 12.47 kV, and 13.8 kV, corresponding to standard feeder designs in North America. Some utilities exhibit bi- or multi-modal patterns, indicating the coexistence of legacy and modern systems. These standardized levels are essential for validating engineering consistency and supporting future circuit upgrades or DER deployments.

### Circuit Rating Distributions

**Fig. 5:**
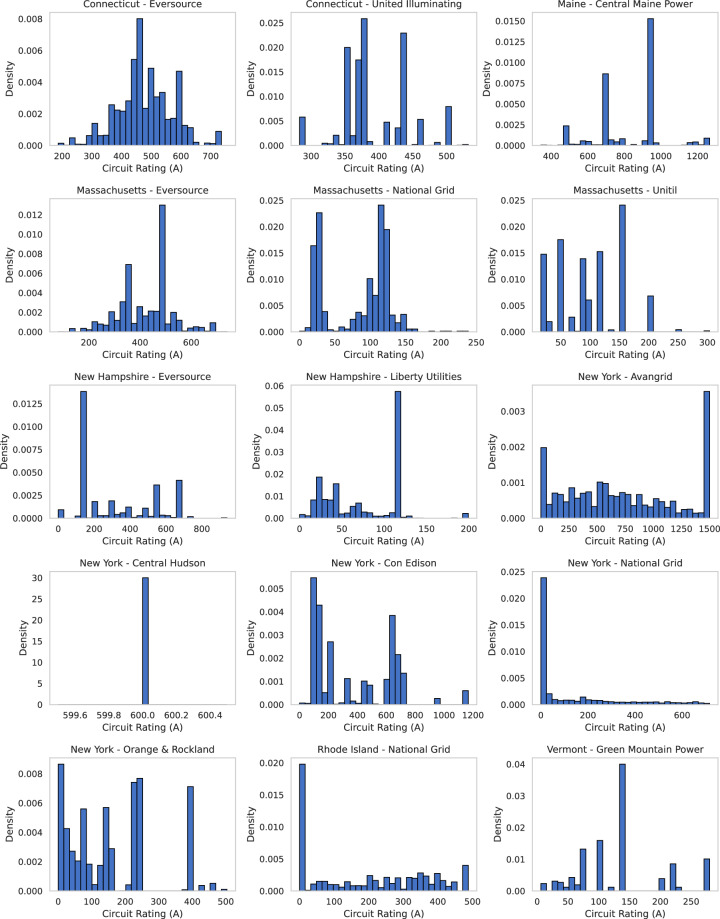
Circuit Rating (A) histograms by utility operating company.

As shown in Fig. 5, circuit current ratings (in Amps) vary significantly by utility, often displaying long-tailed or multimodal distributions. Utilities with larger service areas or diverse customer bases show higher variance, with peaks reflecting feeder design constraints. These ratings are central to estimating power delivery limits and voltage where missing, and their distribution provides a diagnostic tool for identifying atypical segments or data anomalies.

### Goodness-of-Fit Analysis

**Fig. 6:**
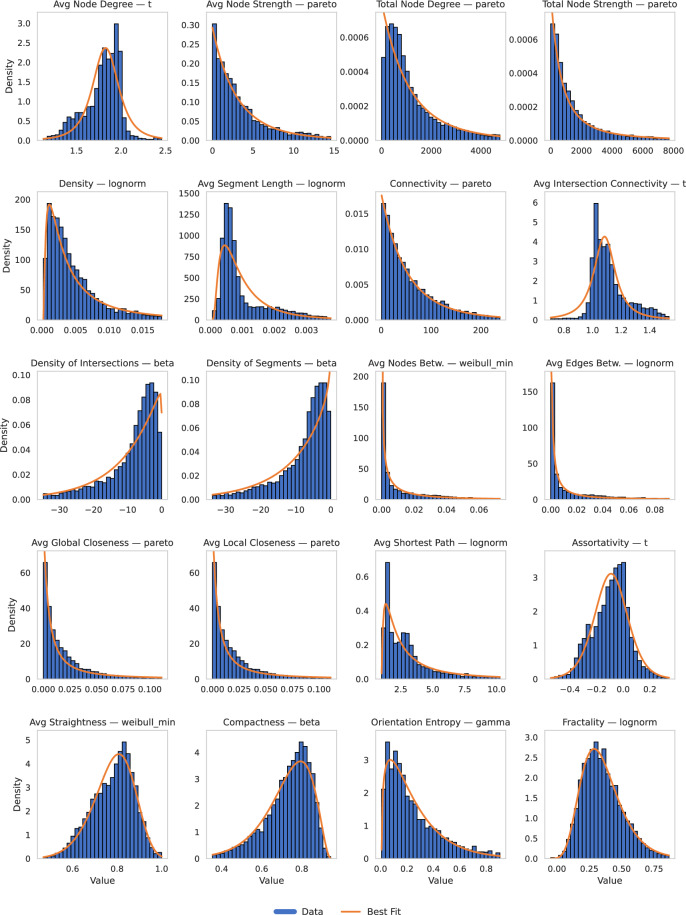
Histograms (blue) and best-fit distributions (orange) for each global topological variable.

To validate each numerical variable’s distributional characteristics, we conducted Kolmogorov-Smirnov (KS) goodness-of-fit tests using a library of 10 candidate continuous distributions: Normal, Log-Normal, Exponential, Gamma, Weibull, Beta, Logistic, Cauchy, Rayleigh, and Uniform. These were selected to capture a broad range of plausible data-generating mechanisms commonly found in physical, engineering, and socioeconomic systems. The results are shown in Table 6. For example, log-normal and gamma distributions are often suitable for skewed variables like circuit ratings or hosting capacities, while Weibull is frequently used in reliability analysis of infrastructure systems. The normal and logistic distributions serve as standard benchmarks for symmetric data, while Cauchy and Rayleigh were included to test for heavy-tailed behavior. For each global metric and each state, we fit all 10 distributions and selected the best-fitting one based on the highest KS p-value. Variables with p > 0.05 were deemed to have acceptable distributional support. In practice, log-normal and gamma distributions emerged as best-fitting for most topological variables (e.g., node strength, edge density, and centrality measures), while normal and logistic distributions occasionally fitted metrics like average segment length or closeness centrality. The results of these fits (shown in Fig. 6) informed log-transformations for subsequent regression modeling and improved interpretability of outlier behavior. For the sake of simplicity we only added to the respository code and this manuscript the overall Hosting Capacity, Operating Voltage, and Circuit Rating distributions, with the functions held in network_analysis.py and visualization.py.

**Table 6:** Best-Fit Distributions for Global Topological Variables.

Variable	Best Fit	KS p-value	Main Parameters (abbreviated)
Avg Node Degree	t	1.84 × 10^−47^	(*df* = 2.76, *loc* = 1.83, *scale* = 0.15)
Avg Node Strength	pareto	2.68 × 10^−3^	(*b* = 6.41, *loc* = − 21.88, *scale* = 21.88)
Total Node Degree	pareto	1.00 × 10^−16^	(*b* = 7.07, *loc* = − 8778.18, *scale* = 8780.18)
Total Node Strength	pareto	7.05 × 10^−5^	(*b* = 1.39, *loc* = − 1589.80, *scale* = 1589.80)
Density	lognorm	3.78 × 10^−12^	(*s* = 1.16, *loc* ≈ 0, *scale* ≈ 0.002)
Avg Segment Length	lognorm	1.06 × 10^−66^	(*s* = 0.82, *loc* ≈ 0, *scale* ≈ 0.003)
Connectivity	pareto	0.109	(*b* = 7.45, *loc* = − 423.22, *scale* = 424.22)
Avg Intersection Connectivity	t	3.17 × 10^−76^	(*df* = 1.72, *loc* = 1.08, *scale* = 0.08)
Density of Intersections	beta	4.68 × 10^−30^	(*a* ≈ 7.81*e*7, *b* ≈ 1.03, *loc* = − 8.18*e*4, *scale* = 8.18*e*4)
Density of Segments	beta	1.09 × 10^−35^	(*a* = 42.43, *b* = 0.92, *loc* = − 497, *scale* = 497)
Avg Nodes Betweenness	weibull_min	1.01 × 10^−14^	(*c* = 0.51, *loc* ≈ 0, *scale* = 0.002)
Avg Edges Betweenness	lognorm	5.23 × 10^−18^	(*s* = 2.22, *loc* ≈ 0, *scale* = 0.004)
Avg Global Closeness	pareto	8.48 × 10^−24^	(*b* = 0.70, *loc* = − 0.0077, *scale* = 0.0077)
Avg Local Closeness	pareto	8.48 × 10^−24^	(*b* = 0.70, *loc* = − 0.0077, *scale* = 0.0077)
Avg Shortest Path	lognorm	1.04 × 10^−15^	(*s* = 1.23, *loc* = 0.98, *scale* = 1.56)
Assortativity	t	6.30 × 10^−10^	(*df* = 9.65, *loc* = − 0.09, *scale* = 0.13)
Avg Straightness Centrality	weibull_min	7.96 × 10^−5^	(*c* = 7.49, *loc* = 0.19, *scale* = 0.63)
Compactness	beta	3.97 × 10^−6^	(*a* ≈ 1.66*e*8, *b* ≈ 3.10, *loc* = − 1.20*e*8, *scale* = 1.20*e*8)
Orientation Entropy	gamma	7.49 × 10^−5^	(*a* = 1.36, *loc* = 0.008, *scale* = 0.18)
Fractality	lognorm	6.09 × 10^−10^	(*s* = 0.31, *loc* = − 0.17, *scale* = 0.50)

## Usage Notes

The dataset is designed to support a wide range of applications in urban planning, infrastructure analysis, and energy equity research. Its structure enables seamless integration into spatial and statistical workflows and allows reproducible network analysis at the census tract level. The dataset includes only technical and topological attributes of the electric distribution network. However, each network segment and census tract is tagged with a GEOID, allowing users to join this dataset with external demographic, economic, or environmental datasets at the tract level.

**Table 7:** Utilities, states, and exact ArcGIS REST endpoints used in this study.

Company	State	Source (REST Endpoint)
Avangrid (CMP)^15^	Maine	https://services.arcgis.com/c0HK6TaWF3mGiNhc/ArcGIS/rest/services/CMP_Hosting_Capacity/FeatureServer
Avangrid (NYSEG/RGE)^16^	New York	https://services.arcgis.com/c0HK6TaWF3mGiNhc/ArcGIS/rest/services/HostingCapacity_DCirc_NYSEG_RGE/FeatureServer
Central Hudson^17^	New York	https://services1.arcgis.com/CEN9MBRF2dIzEmKF/ArcGIS/rest/services/Hosting_Capacity_Stage3/FeatureServer
Con Edison^18^	New York	https://services.arcgis.com/ciPnsNFi1JLWVjva/ArcGIS/rest/services/CECONY_NodalHCV_Prod/FeatureServer
Eversource^19^	Connecticut	https://dghostingexternalmapprod.eversource.com/dghosting/rest/services/DG_Hosting_Final
Eversource^20^	Massachusetts	https://dghostingexternalmapprod.eversource.com/dghosting/rest/services/DG_Hosting_Final
Eversource^21^	New Hampshire	https://dghostingexternalmapprod.eversource.com/dghosting/rest/services/DG_Hosting_Final
Green Mountain Power^22^	Vermont	https://maps.gmpvt.com/arcgis/rest/services/CapacityPlanningMap/MapServer
Liberty Utilities^23^	New Hampshire	https://services5.arcgis.com/YAfU4W3mlwAI585a/ArcGIS/rest/services
National Grid^24^	Massachusetts	https://systemdataportal.nationalgrid.com/arcgis/rest/services/MASDP/MASDP_HostingCapacity/MapServer/
National Grid^25^	New York	https://systemdataportal.nationalgrid.com/arcgis/rest/services/NYSDP/Hosting_Capacity_Data/MapServer
Orange & Rockland (ORU)^26^	New York	https://services.arcgis.com/ciPnsNFi1JLWVjva/arcgis/rest/services/ORU_NodalHCV_Prod/FeatureServer/
United Illuminating (Avangrid)^27^	Connecticut	https://services1.arcgis.com/KUQsV0ju95o9UMmS/arcgis/rest/services/UI_DG_HC/FeatureServer
Unitil^28^	Massachusetts	https://gisdata.unitil.com/arcgis/rest/services/Electric/HostCapacityConductorCircuits/MapServer/8
Unitil^29^	New Hampshire	https://gisdata.unitil.com/arcgis/rest/services/Electric/HostCapacityNH/MapServer/8

### Data Access and Formats

The core dataset is provided in open formats, such as Shapefiles (.shp) for electric circuit segments with geospatial attributes or CSV files (.csv) for node-level and tract-level topological metrics. These files can be opened using common GIS software (e.g., QGIS, ArcGIS) or read directly into Python using GeoPandas and Pandas.

### Software and Processing

All processing is implemented in Python 3.10+ and designed for execution in a Conda environment. The main libraries include geopandas for geospatial operations; networkx, momepy, and shapely for network analysis; pandas and numpy for data manipulation and aggregation; matplotlib and seaborn for data visualization. The pipeline is modular and includes scripts for data cleaning, network construction, metric calculation, and figure generation.

### Getting Started

All steps are documented in the repository’s README.md file. The unprocessed and cleaned data files are stored in the Harvard Dataverse repository at the following URL: https://dataverse.harvard.edu/dataverse/us_electric_networks.

The following is a quick guide to reproduce the dataset or extend the analysis.

1. Clone the repository from https://github.com/bernatsalbanya/US-Electric-Distribution-Networks.git and navigate into the folder.
2. (Optional) Create a virtual environment with conda create –name US-Electric-Distribution-Networks python=3.10 and activate it.
3. Install dependencies using either python setup.py build and python setup.py install, or pip install -r requirements.txt
4. Modify file paths and parameters in config.yaml as needed.
5. Navigate to the scripts folder and run the main pipeline python main.py -h for help, python main.py -p -d to download, preprocess and analyze data, python main.py -p to preprocess and analyze data, or python main.py to analyze cleaned data only.

### Applications

Potential applications of this dataset include optimization of the electric grid through quantitative analysis of the topology, redundancy, and vulnerability of distribution networks; performing a full or simplified power flow simulation; energy equity analysis by enabling integration with external indicators of energy burden or income inequality; urban infrastructure planning to support data-driven decisions for grid modernization and electrification strategies; and machine learning and clustering, such as training models to classify or forecast network performance based on topological metrics.

## Data Availability

All datasets generated in this study are publicly available in the US Electric Distribution Networks Dataverse. The raw circuit-level data are deposited under accession code^31^ (10.7910/DVN/HSHLLT). The cleaned and harmonized dataset, including the integrated shapefile US_Northeast_Electric_Network.shp, is available under accession code^30^ (10.7910/DVN/8Z4JRD). The Census tract geometries used as the spatial unit of analysis are archived under accession code^33^ (10.7910/DVN/XZBDDR). The results repository, containing network edge geometries, node-level metrics, and census-tract-level global indicators, is available under accession code^32^ (10.7910/DVN/PPL4JZ). All files are provided in open formats (.shp for spatial data and .csv for tabular data) with persistent DOIs to ensure reproducibility and transparency.

## Code availability

All code used to generate, clean, merge, and analyze the electric distribution dataset is publicly available in the following GitHub repository: https://github.com/bernatsalbanya/US-Electric-Distribution-Networks. The repository is released under the MIT license and includes all scripts, configuration files, and documentation necessary for full pipeline reproducibility. All scripts were tested using Python 3.10 in a Conda-managed environment. Versioning and usage instructions are included in the repository’s README.md.
